# Primary Cutaneous B-Cell Lymphomas: An Update

**DOI:** 10.3389/fonc.2020.00651

**Published:** 2020-05-27

**Authors:** Paola Vitiello, Antonello Sica, Andrea Ronchi, Stefano Caccavale, Renato Franco, Giuseppe Argenziano

**Affiliations:** ^1^Dermatology Unit, University of Campania Luigi Vanvitelli, Naples, Italy; ^2^Department of Precision Medicine, University of Campania Luigi Vanvitelli, Naples, Italy; ^3^Pathology Unit, Department of Mental and Physical Health and Preventive Medicine, University of Campania Luigi Vanvitelli, Naples, Italy

**Keywords:** primary cutaneous marginal zone lymphoma, primary cutaneous follicle-center cell lymphoma, diffuse large B-cell lymphoma, leg type, intravascular large B-cell lymphoma, EBV-positive mucocutaneous ulcer

## Abstract

Primary cutaneous B-cell lymphomas (PCBCLs) comprise a group of extranodal B-cell non-Hodgkin lymphomas B-cell derived, which primarily involve the skin without evidence of extracutaneous disease at the time of diagnosis. They include ~25% of all cutaneous lymphomas and are classified in three major subgroups (World Health Organization (WHO) 2017): primary cutaneous marginal zone lymphoma (PCMZL), primary cutaneous follicle-center cell lymphoma (PCFCL), and diffuse large B-cell lymphoma, leg type (PCDLBCL, LT). This classification also includes some less common entities such as intravascular large B-cell lymphoma. Recently, WHO-EORTC added Epstein–Barr virus positive (EBV+) mucocutaneous ulcer, as a new provisional distinct entity, to cutaneous B-cell lymphomas. PCBCLs are classically characterized by patches, plaques, or nodules showing great variability for color, shape, and location. Diagnosis requires histological examination with immunohistochemical staining. In general, therapeutic options depend on the exact histological and immunohistochemical classification, disease presentation, and risk assessment. PCMZL and PCFCL are considered indolent lymphomas with a good prognosis and are associated with 5-year disease-specific survival ≥ 95%. In contrast, PCDLBCL, LT is considered an aggressive lymphoma with a survival rate in 5 years of lower than 60%. Patients with a solitary lesion or limited lesions in a single anatomical site require different treatments as compared to patients with generalized lesions or refractory disease or extracutaneous involvement. Therapeutic choice includes observation, local, or systemic therapy based on histology and disease extension. Patient management is multidisciplinary, including dermatologists, pathologists, hemato-oncologists, and radiation oncologists.

## Introduction

PCBCLs comprise a group of extranodal B-cell non-Hodgkin lymphomas that primarily involve the skin without evidence of extracutaneous disease at the time of diagnosis. They include ~25% of all cutaneous lymphomas and are classified in three major subgroups (WHO 2017): primary cutaneous marginal zone lymphoma (PCMZL), primary cutaneous follicle center lymphoma (PCFCL), and diffuse large B-cell lymphoma, leg type (PCDLBCL, LT) (Table 1). This classification also includes some less common entities such as intravascular large B-cell lymphoma. Recently, WHO-EORTC added Epstein–Barr virus positive (EBV+) mucocutaneous ulcer, as a new provisional distinct entity, to cutaneous B-cell lymphomas. The incidence of PCBCLs has been increasing over the decades and is currently about 4 per million persons. Male patients, non-Hispanic whites, and adults over 50 years of age are mainly affected. Excisional or punch skin biopsy, with histological and immunohistochemical analysis, represents a key role in the diagnosis of PCBCL (1–5). Dermoscopy has recently focused on the diagnosis of cutaneous lymphomas and some patterns have been described. The most frequent features are white circles with a salmon-colored background/area, scales, and arborizing/serpentine vessels. These dermoscopic findings are not specific and a correlation between histology and dermoscopy has not yet been found. However, dermoscopy can be useful as an ancillary tool in PCBCLs, and it can assist in the early diagnosis if integrated with clinical manifestations (6, 7). Careful staging, in addition to history and physical examination, are necessary to recognize any extracutaneous involvement and to evaluate timely treatment. A complete blood count, a comprehensive chemistry panel, and lactate dehydrogenase level (LDH) are the recommended laboratory tests for diagnostic study. In addition, PET/CT or CT total body scan with contrast should be performed at diagnosis. In general, therapeutic options depend on the exact histological and immunohistochemical classification and disease presentation, and on the risk assessment. PCMZL and PCFCL are considered indolent lymphomas with a good prognosis and are associated with 5-year disease-specific survival of ≥95%. In contrast, PCDLBCL, LT is considered an aggressive lymphoma with a survival rate in 5 years of lower than 60%. Patients with a solitary lesion or limited lesions in a single anatomical site require different treatments if compared with patients with generalized lesions or refractory disease or extracutaneous involvement. Treatment recommendations for PCBCL are based on small retrospective studies. No randomized controlled trials are available. Therapeutic choice includes observation, local, or systemic therapy based on histology and disease extension. Patient management is multidisciplinary, including dermatologists, pathologists, hemato-oncologists, and radiation oncologists (8–10).

**Table 1:** Quick snapshot of PCBCL.

**Histological Type**	**Presentation**	**Most common involved sites**	**Behavior**	**Immunohistochemical**	**Treatment**
PCMZL	Red-violaceous small solitary or multiple papules or nodules and rarely plaques	Trunk, arms or head	Indolent	CD20 +, CD79a +, BCL2 +, CD5-, CD10-, BCL 6-, MUM 1 -	Radiotherapy or surgical excision, topical drugs, intralesional therapies, immunochemotherapy
PCFCL	Solitary or grouped erythematous or erythemato-violaceous papules, plaques, and/or nodules	Trunk, head or neck	Indolent	CD20+, CD79a+, CD5-, CD10+/-, BCL 6+, BCL2-, MUM-1/IRF-4 negative	Radiotherapy or surgical excision, topical drugs, intralesional therapies, immunochemotherapy
PCDLBCL	Erythemato-cyanotic plaques and/or nodules with rapid growth	Legs	Aggressive	CD20+, CD79a+, BCL2+, CD10-, BCL 6+/-, FOX-P1 and MUM-1/IRF-4 positive	Local radiotherapy, R-CHOP, pegylated liposomal doxorubicin, monoclonal antibodies
IVBCL	Violaceous patches and plaque, painful blue-red nodules, ulcerated tumors or telangiectasic skin lesions	SNC, lungs, and skin	Aggressive	CD20 +, BCL 2+, IRF4/MUM-1 + (MIB-1/Ki 67++)	Chemotherapy in combination with rituximab
EBV-MCU	Solitary, sharply demarcated ulcerating lesion	Oropharyngeal mucosa, skin and gastrointestinal tract	Indolent	Variable expression of CD20; CD19+, CD79a +, CD10-, CD30+, BCL2+, PAX 5+, BCL 6 -, MUM-1/IRF-4+	Local radiotherapy or surgical excision, systemic chemotherapy, rituximab

## Primary Cutaneous Marginal Zone Lymphoma

**Figure 1:**
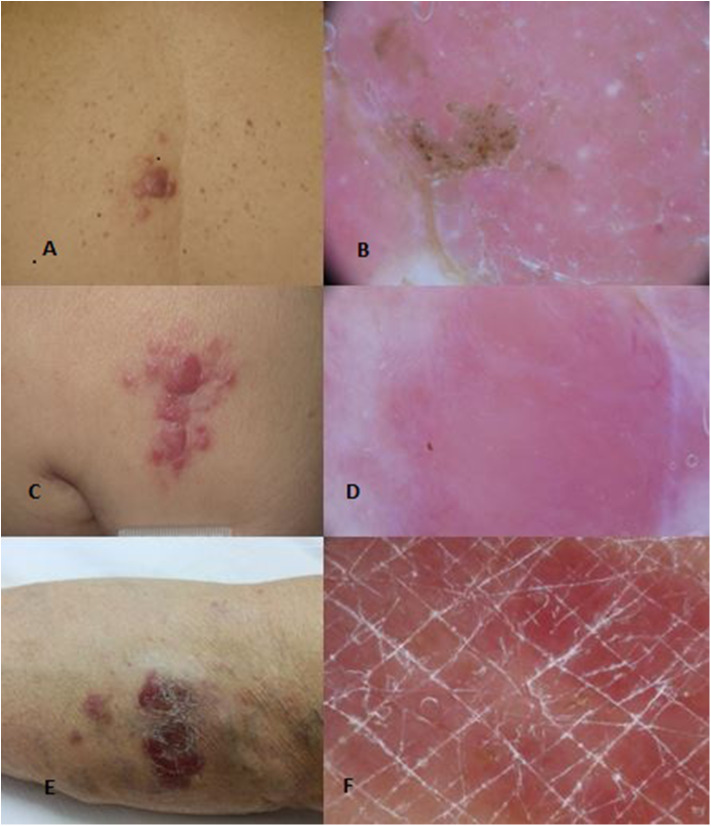
Clinical **(A)** and dermoscopical **(B)** images of a PCMZL. Clinical **(C)** and dermoscopical **(D)** images of a PCFCL. Clinical **(E)** and dermoscopical **(F)** images of a PCDLBCL leg type.

PCMZL accounts for 2–7% of all primary cutaneous lymphomas and about 10% of all marginal zone lymphomas are primary cutaneous forms. PCMZL usually occurs with red-violaceous, small, solitary, or multiple papules or nodules and rarely plaques with very indolent course, preferentially located on the trunk, arms, and occasionally the head (Figures 1A,B). Systemic symptoms are usually absent and the presence of a serum or urinary monoclonal component (MC) represents a very rare occurrence (11–13). Some authors have proposed the distinction of two subsets of PCMZL. The first is characterized by periadnexial and perivascular nodular infiltrate of plasma cells, many intermingled T cells, and absence of CXCR3 expression; this is the most common subtype. The second is characterized by larger nodular infiltrates of neoplastic B cells expressing CXCR3, IGM, and a few reactive T cells (14). Although an etiological link with Borrelia Burgdorferi has been proposed in European patients, several studies have not shown a real etiological correlation. The median age at diagnosis is 55 years. Skin relapses affect up to 50% of patients, while extracutaneous spread is extremely rare. Prognosis is excellent with a 5-year survival rate of 95–100%. Transformation of PCMZL into another type of high-grade lymphoma is possible, and it represents a negative prognostic factor. Blastic transformation of PCMZL is very rare, and the prognosis worsens when it happens.

**Figure 2:**
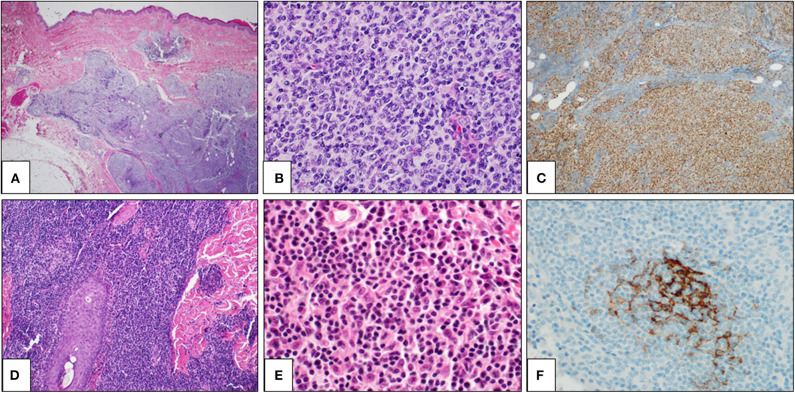
Primary cutaneous germinal center cell lymphoma: histological findings. Histological examination showing a dense lymphoid infiltrate in dermis and hypodermis, organized in vaguely defined nodules. The lymphoid population infiltrates the hypodermis, entrapping adipocytes (**A**: H&E, 2×). The lymphoid nodules are constituted by large and irregular germinal centers (**B**: H&E, 40×) and are positive for bcl6 immunostaining (**C**: bcl6 immunostain, 10×). Primary cutaneous marginal zone lymphoma: histological findings. A diffuse lymphoid population in the reticular dermis, extending along a hair (**D**: H&E, 2×). The lymphoid population is heterogeneous, including mature lymphocytes, lympho-plasmacytoid cells, and plasma cells (**E**: H&E, 40×). CD21 immunostaining highlights a partially destroyed network of follicular dendritic cells (**F**: CD21 immunostain, 40×).

Histologically, PCMZL is characterized by a dense dermal lymphoid infiltrate, which is most often arranged in a vaguely nodular or diffuse pattern (Figure 2D). The infiltrate usually fulfills the dermis and extends to the hypodermis. A Grenz zone is usually evident in the papillary dermis and epidermal ulceration is absent. The cellular population is quite heterogeneous, including small- to medium-sized B cells and centrocyte-like cells, with lymphoplasmacytic cells, monocytoid cells, and plasma cells (Figure 2E). Some reactive germinal centers are usually evident, but not always present. Although the lymphoid infiltrate tends to spread around the dermal adnexa, lympho-epithelial lesions are rare. When present, follicular colonization by marginal zone cells is a diagnostic clue. Immunohistochemically, the neoplastic cells are CD20+, CD79a+, bcl2 + CD5-, CD10-, and BCL6- (Figure 2F). Differential diagnosis with cutaneous B-cell pseudolymphoma is difficult, and molecular tests are often needed to demonstrate the clonal rearrangement of the immunoglobulin heavy chain (IgH) gene. Contrary to what is reported in nodal marginal zone lymphoma, genetic translocations are rare, as t_(14;18)_, t_(11;18)_, and t_(3;14)_ have been reported only in 25, 7, and 10% of PCMZLs, respectively.

According to EORTC/ISCL consensus recommendation, patients with solitary or few contiguous lesions can be treated with radiotherapy or surgical excision with curative intent. Several studies propose the use of low-dose RT (LDRT) (4 Gy) because it has shown high response rates by reducing the rates of acute toxicity (14). Antibiotic treatment is required in Borrelia antibody-positive cases of PCMZL. Many topical therapies are available with good response: clobetasol, nitrogen mustard, cryotherapy, and imiquimod. Intralesional treatment with triamcinolone or interferon α (INFα) or rituximab can be used as a second-line treatment or in patients with multiple localized lesions (15). In these cases, intravenous rituximab (16), chlorambucil, or local radiotherapy can also be used. In PCMZL such as in all indolent lymphomas, a wait-and-see strategy can be followed. This strategy consists of observing and treating the patient only when the disease shows systemic symptoms, bulky mass (<7 cm), symptomatic splenomegaly, progressive leukemization, and serum effusions. Follow-up is usually planned every 6 months with a complete skin and lymph node examination (17–21).

## Primary Cutaneous Follicle Center Lymphoma

Primary cutaneous follicle center lymphoma (PCFCL) is an indolent lymphoma that presents with solitary or, less commonly, grouped erythematous or violaceous papules, plaques, and/or nodules with predominantly loco-regional distribution, and it shows a preference for the trunk and head–neck district (Figures 1C,D). Ulcerations are rarely observed. PCFCL most commonly affects middle-aged patients with a slight male predominance and represents ~55–60% of all PCBCLs. The prognosis is excellent with a 5-year disease-specific survival of 95%. Without treatment, lesions may be stable or enlarge slowly often after a few years. In rare cases, lesions may also spontaneously regress. Histologically, PCFCL is characterized by a dermal infiltrate organized into three different patterns depending on the type of skin lesion and its onset: follicular, follicular and diffuse, and diffuse. These growth patterns do not differ in behavior or prognosis. In papules and plaques of recent onset, there is a follicular or follicular and diffuse pattern. This pattern can be a common diagnostic pitfall of pseudolymphoma. Subsequently, the infiltrate becomes more widespread and follicular structures are no longer visible. The lymphoid cells are constituted by a variable account of small centrocyte-like cells and large centroblast-like cells F (Figure 2A). The phenotypes of cancer cells are CD20+, CD79a+, CD5−, CD10+/−, bcl 6+, and MUM-1/IRF-4 negative (Figures 2B,C). Importantly, bcl 2—which is one of the immunohistochemical clues of nodal follicular lymphoma—is most often negative. Moreover, the majority of patients do not harbor the t_(14;18)_ translocation involving bcl-2 locus, in contrast to systemic follicular lymphoma. Clonally rearranged immunoglobulin genes are present in most cases. The differential diagnosis includes acne, cysts, arthropod bites, basal cell carcinoma, Merkel cell carcinoma, other non-B-cell cutaneous lymphomas (22), and cutaneous lymphoid hyperplasia. Bone marrow examination should be considered. According to Senff et al., legs as a presentation site influence the prognosis, with an overall survival of 41% at 5 years [71-72]; coexistence with DLBCL also worsens the prognosis of PCFCL (23, 24). Transformation of PCFCL into DLBCL is not very rare and together with transformations into another type of high-grade lymphoma represents negative prognostic factors. Solitary lesions require radiation therapy or complete surgical excisions with curative intent. Several authors recommend the use of LDRT (4 Gy) for their better outcome and reduced toxicity in particular in all indolent forms (24). Intralesional (e.g., corticosteroids or rituximab) or topical therapies (e.g., corticosteroids, nitrogen mustard, imiquimod, cryotherapy) may also be considered. For patients with multiple localized lesions, radiotherapy in multiple radiation fields can also be practiced, whereas in patients with exclusively scattered skin lesions, the recommended behavior is to watch and wait treating only symptomatic lesions. For generalized skin lesions, systemic administration of rituximab is an effective treatment. Rarely patients with PCFCL develop extracutaneous disease, and multiagent chemotherapy (R-CHOP) represents a useful therapeutic option. Cutaneous recurrences are frequently observed (30–46.5%), usually confined to the skin. Follow-up intervals are appropriate every 6 months and consist of a complete cutaneous and nodal clinical examination (23, 24).

## Primary Cutaneous Diffuse Large B-Cell Lymphoma, Leg Type

Primary cutaneous diffuse large B-cell lymphoma, leg type (PCDLBCL, LT) is a rare but aggressive lymphoma with a poor outcome and lower complete response rates to therapy. It is characterized by erythemato-cyanotic nodules and/or plaques with a rapid growth, very often located in correspondence of one or both legs and only in 15–20% of patients in different sites (Figures 1E,F). PCDLBCL, LT shows tendency to extracutaneous diffusion with a more unfavorable prognosis. Patients can present “B-symptoms” (fever, night sweats, and weight loss). Death is not uncommon, and the rate of 5-year survival is ~50%. It represents 20% of all PCBCLs, and it most commonly affects elderly woman (2:1 ratio over men). The histological picture is characterized by a diffuse dermal infiltrate composed largely of a monotonous population of centroblasts and immunoblasts, with high mitotic activity and minimal reactive T component. The phenotypes of cancer cells are CD20+, CD79a+, bcl2+, CD10–, bcl 6+/–, FOX-P1, and MUM-1/IRF-4 positive. Monoclonal rearrangement for immunoglobulin heavy chains is constantly present. Translocations, deletions, and mutations that could be useful in diagnosing the disease have been recently identified. A bone marrow biopsy is recommended. Differential diagnosis usually includes other systemic NHLs, leukemia, and other neoplastic diseases (25–27). PCDLBCL leads to a greater number of relapses, and it has a shorter time to progression compared to indolent lymphomas. The localization on the leg, the presence of multiple skin localizations, the round cell morphology, the high expression of MUM1 and FOX-P1, and the deletion of the CDKN2A locus on chromosome 9p21 seem to be negative prognostic factors (28). In patients with single lesion, local radiation therapy is the choice with the possible addition of R-CHOP. Like for all lymphomas, before starting each chemotherapy protocol, it is good practice to search for HCVAb, HBsAg, and HBcAb to check for any viral reactivation and prevent it with the indicated prophylaxis (28–32). Patients with generalized lesions are treated as in nodal DLBCLs. Promising results have been obtained from clinical trials with bortezomib, lenalidomide, and ibrutinib. In more aggressive cases with contraindications to R-CHOP-like regimens, the association of rituximab with pegylated liposomal doxorubicin may be an alternative approach. Two monoclonal antibodies, lumilixumab and dacetuzumab, are in clinical trials for future treatments. Follow-up intervals are usually performed every 3 months after initial treatment. Every follow-up consists of a complete cutaneous and nodal clinical examination in laboratory and PET CT evaluation and annual restaging (33–40). After the end of the therapy, PET CT scan is needed every 6 to 12 months.

## Intravascular Large B-Cell Lymphoma

Intravascular large B-cell lymphoma (IVBCL) is a rare subtype of large B-cell lymphoma presenting with violaceous patches and plaques, painful blue-red nodules, ulcerated tumors, or telangiectasic skin lesions. All lesions preferentially involve central nervous system, lungs, and skin. This lymphoma subtype is typically characterized by an accumulation of large neoplastic B cells within blood vessels, capillaries, small arteries, and small veins. Angiodestruction is rare. The phenotypes of cancer cells are CD20+, BCL2+, and IRF4/MUM-1+ with active proliferation (MIB-1/Ki 67++). Clonal immunoglobulin rearrangement is usually present. It was first described in 1959 as angioendotheliomatous proliferans. Patients often have widely disseminated disease. Rare cases with only the skin involved have been reported, whereas most common cases involve the legs and trunk. The prognosis is poor: 3-year overall survival for 56% of patients with only cutaneous lesions and 22% for patients with widespread disease. Patients may present with symptoms related to the involved organ, accompanied by an elevated erythrocyte sedimentation rate and an elevated LDH, or may be asymptomatic. Normochromic normocytic anemia is typically found. Currently multiagent chemotherapy in combination with rituximab is the preferred treatment. Additionally, CNS-directed therapy with methotrexate should be considered in patients with neurological involvement (41). In patients with diffuse disease and worse prognosis, it is important to consider adequate therapy for pain control (42).

## EBV-Positive Mucocutaneous Ulcer (EBV-MCU)

In immunosuppressed patients, EBV can elicit B-cell transformation and proliferation by exploiting reduced immune surveillance. EBV-positive mucocutaneous ulcer (EBV-MCU) is a rare B-cell proliferation initially described in 2003 by Oyama and has been included as a new provisional entity in the revised WHO classification of 2016. Among the patients generally affected, we can find bone marrow or solid organ transplant recipients and with age-related or iatrogenic immunodeficiency. EBV-MCU is characterized by a solitary, sharply demarcated ulcerating lesion and has a self-limited, indolent course. The ulcers involve oropharyngeal mucosa, skin, and gastrointestinal tract. Lesions show a polymorphous infiltrate with a mixture of lymphocytes, immunoblasts, and large transformed B cells often with Hodgkin/Reed–Sternberg cell-like morphology. The phenotype of cancer cells shows variable expression of CD20 and presents CD19+, CD79a+, CD10-, BCL2+, PAX5+, BCL6-, and MUM-1/IRF-4+. Coexpression of CD30 with CD15 is found in almost half of the cases. EBV-MCU usually has an indolent, self-limited course. When possible, reduction of immunosuppressors is the first treatment. A conservative management, including rituximab or other specific treatment against EBV, is appropriate (43).

## Discussion and Conclusion

PCBCLs represent a disease in which there is certainly still much to investigate and that requires a careful diagnostic and staging process, which is essential for patient management. The current focus on genetic and epigenetic mechanisms that characterize lymphoproliferative processes and the search for new markers could open up new classification and diagnostic–therapeutic perspectives (44). The current subdivision of primitive cutaneous B-cell lymphomas into indolent and aggressive forms guides the appropriate therapeutic choice. The therapeutic indications, however, are based on retrospective and small cohort studies of patients often with short follow-up periods, which represents a limit for long-term patient management. PCMZL and PCFCL are indolent diseases, which should be treated with radiotherapy, surgery, or intralesional steroids. Overtreatment should be avoided. Multiagent chemotherapy should be reserved for patients developing extracutaneous disease or with widespread and nonresponsive skin lesions (45). PCDLBCL, LT should preferentially be treated as other diffuse large B-cell lymphoma, with CHOP-like regimens associated with rituximab and local radiotherapy (46–48). It is recommended that the patient is followed by a multidisciplinary team of experts in the management of these disorders for optimal treatment and careful follow-up.

## Author Contributions

AS, PV, and AR: conceptualization, writing-review and editing, and data curation and investigation. AS, PV, AR, and SC: methodology. AS, PV, AR, GA, and RF: project administration. PV: writing-original draft. All authors have read and agreed to the published version of the manuscript.

## Conflict of Interest

The authors declare that the research was conducted in the absence of any commercial or financial relationships that could be construed as a potential conflict of interest.
